# Understanding Immunology via Engineering Design: The Role of Mathematical Prototyping

**DOI:** 10.1155/2012/676015

**Published:** 2012-09-03

**Authors:** David J. Klinke, Qing Wang

**Affiliations:** ^1^Department of Chemical Engineering and Mary Babb Randolph Cancer Center, West Virginia University, Morgantown, WV 25606, USA; ^2^Department of Microbiology, Immunology and Cell Biology, West Virginia University, Morgantown, WV 25606, USA; ^3^Department of Computer Sciences, Mathematics, and Engineering, Shepherd University, Shepherdstown, WV 25433, USA

## Abstract

A major challenge in immunology is how to translate data into knowledge given the inherent complexity and dynamics of human physiology. Both the physiology and engineering communities have rich histories in applying computational approaches to translate data obtained from complex systems into knowledge of system behavior. However, there are some differences in how disciplines approach problems. By referring to mathematical models as mathematical prototypes, we aim to highlight aspects related to the process (i.e., prototyping) rather than the product (i.e., the model). The objective of this paper is to review how two related engineering concepts, specifically prototyping and “fitness for use,” can be applied to overcome the pressing challenge in translating data into improved knowledge of basic immunology that can be used to improve therapies for disease. These concepts are illustrated using two immunology-related examples. The prototypes presented focus on the beta cell mass at the onset of type 1 diabetes and the dynamics of dendritic cells in the lung. This paper is intended to illustrate some of the nuances associated with applying mathematical modeling to improve understanding of the dynamics of disease progression in humans.

## 1. Introduction

**Figure 1:**
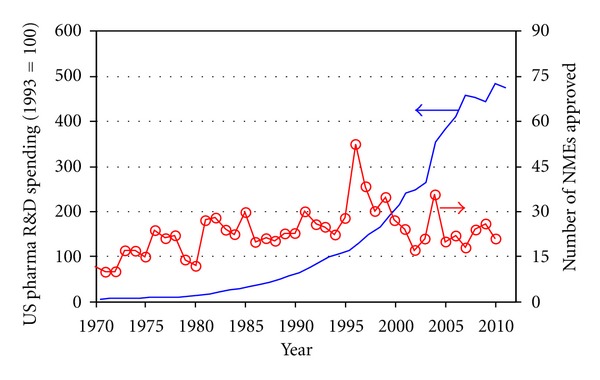
Productivity metrics of the United States pharmaceutical industry. Research and development spending by the United States pharmaceutical industry has escalated dramatically during the last several decades (solid line—left axis) [5]. However, the translation of this increased research spending into new therapeutic products, as represented by the number of new medical entities (NMEs) approved by the Food and Drug Administration (circles—right axis), has failed to keep pace [2].

 One of the great challenges in the field of health science is understanding how to integrate the knowledge obtained about individual molecules and cells to predict integrated system behavior [1]. Advances in the techniques associated with molecular biology during the twentieth century provided immense insight into the individual components of complex biological systems. Integration of this new technology has also changed the nature of immunological research—from static single measurements to large-scale data-intensive assays obtained at multiple time points. As highlighted in Figure 1, research costs associated with these new techniques have escalated dramatically, but the commercialization rate of new therapeutic products has been unable to keep pace [2]. This increasing disconnect between cost and commercialization also corresponds to a growing awareness of the need to improve understanding of how the identified biological parts function together in biological systems and how dysfunction manifests itself as disease [3, 4].

Historically, engineering is an applied field in which knowledge of how components of a system work, which is obtained through basic research, is synthesized into commercially viable products and processes. A fundamental pillar in this field is the use of computational frameworks for interpreting and predicting the behavior of complex systems [6]. These computational frameworks integrate fragmented knowledge and enable one to explore novel experimental conditions, as a type of in silico screening. By recreating a real system in silico, the predictive power of the simulation (or lack thereof) may be used to infer hidden components or unknown relationships among existing ones. Engineering can provide value to the drug development process by translating observations of the state of a system, that is, experimental data, into quantitative knowledge about how biological systems work. In particular, this approach can aid in understanding the implications of dynamic relationship among biological components of a system and can identify knowledge gaps in the collective understanding of a biological system.

Interestingly, parallels can be drawn between the development of the modern experimental techniques of molecular biology and the advances in experimental chemistry during the middle part of the 20th century. These advances in experimental chemistry were critical driving forces for the emergence of modern chemical engineering [7]. During this period, modern chemical engineering played a central role in developing computational tools that helped transform chemistry from a qualitative into a predictive science. More recently, chemical engineering is evolving to incorporate molecular biology as another enabling science, in addition to physics and chemistry [8]. Our increased ability to probe the molecular basis for cellular response provides an intriguing context for applying engineering principles, such as thermodynamics, transport phenomena, chemical kinetics, and multiscale analysis. From the biology perspective, the National Research Council in the United States identified a need for deeper integration of theory into biological research [9]. All immunologists, to some extent, act as theorists in designing and interpreting experiments. However in this context, theory is encoded in a computable form that facilitates quantitative validation of the theory against data. In fact, mathematical approaches have a rich history in physiology (e.g., [10]). Computational frameworks are also used quite extensively in engineering for interpreting and predicting the behavior of complex systems [6]. However, there are some nuances associated with mathematical modeling within the context of the engineering discipline that may be helpful outside of the discipline. One of the challenges facing the integration of engineering approaches into the drug development process is that there is little understanding of what engineers actually do [11]. To help bridge that gap, the objective of this paper is to review how two related concepts in engineering, namely prototyping and “fitness for use”, are applied to improve understanding of immunology in the context of human physiology.

## 2. What Are Prototyping and “Fitness for Use”?

**Figure 2:**
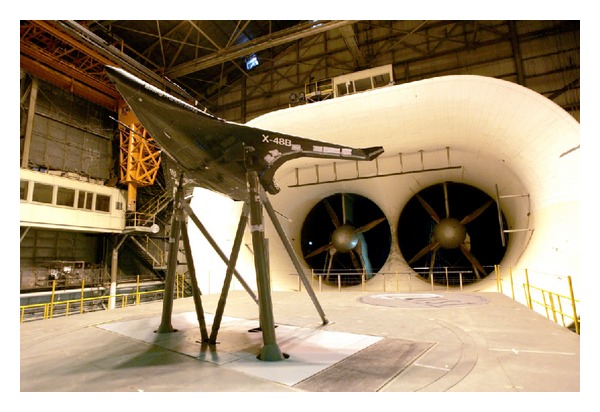
A common example of a prototype. A prototype of a blended wing body aircraft, the X-48B, is shown in a wind tunnel at NASA's research center in Langley Air Force Base, VA. The wind tunnel was used by researchers to evaluate this prototype against structural, aerodynamic, and operational design objectives for an advanced aircraft concept (NASA photo/Jeff Caplan).

 Engineers synthesize scientific and mathematical knowledge to solve problems using an iterative process called engineering design. A traditional application of engineering design includes developing a physical representation of the solution in the form of a prototype, such as a scale-model of an aircraft for use in a wind tunnel (see Figure 2). However, our knowledge of the system of interest is invariably uncertain. Uncertainties create options in the design process that one must select among. Prototypes developed at intermediate stages during the design process can represent alternative solutions and thereby provide a mechanism for making informed decisions. Informed decisions during the design process guide researchers iteratively towards a global solution to all of the design objectives. Collectively, the engineering design process is a knowledge generating activity [12]. Thus, these prototypes provide an essential role by improving the understanding of the problem, by identifying gaps in knowledge (i.e., uncertainties), by soliciting feedback from end users, and by providing a mechanism to evaluate the fitness of the solution against design objectives [13]. It is this last role that relates to the term “fitness for use.” Fitness for use is used to characterize how well an object fulfills its intended purpose, no more or no less [14]. Details that have no influence on fitness of the solution can be removed from consideration. Conversely, clarity about the intended purpose is required prior to creating a prototype. It is this iterative back and forth between clarifying the purpose and creating the prototype that enables reaching an optimal succinct solution.

Prototypes can also include nonphysical objects, such as a mathematical model. A mathematical model is a complete and consistent set of mathematical equations that describe the behavior of the system of interest [15]. The equations represent an explicit external description of a mental solution to the problem of synthesizing new knowledge from inspecting data. The process of constructing a mathematical model forces the researcher to wrestle with these same engineering design concepts (e.g., problem definition, uncertainties, feedback, and fitness). Mathematical models can be particularly valuable in drug discovery by improving the understanding of the problem and by identifying uncertainties in domain knowledge relevant to the target of interest. How uncertainties influence the ability of a prototype to achieve the design objectives can be quantified using well-defined techniques, such as sensitivity analysis [16] or empirical Bayesian approaches for model-based inference [17].

Interdisciplinary work can also be facilitated by using “boundary” objects that reside between two different cultures [18], such as engineering and health science. A mathematical model, as a type of boundary object, imposes formalism by requiring an explicit account of the interacting elements and their relationships. In addition, boundary objects facilitate common understanding through debate and building consensus with regard to what should be included or excluded from the model. By explicitly representing knowledge associated with different scientific domains, the process of modeling can also help improve problem definition.

In essence, the primary goal of making a mathematical model is to make predictions: what do we expect to happen in a particular interacting system under particular conditions, given our current understanding of interactions among components of the system? Similarities between the simulated behaviors and observed data confirm our explicit statements while differences highlight areas of uncertainty in our understanding and provide the engine for scientific progress [19]. By referring to mathematical models as mathematical prototypes, it is the process that one uses to generate the model (i.e., prototyping) that we are intending to highlight rather than the product (i.e., a mathematical model). In the following sections, two examples are presented where a mathematical prototype that was created to address questions related to type 1 diabetes and the role of dendritic cells in adaptive immunity.

## 3. Example 1: Beta Cell Mass and Onset of ****Type 1 Diabetes

 Type 1 diabetes mellitus is characterized by an impaired ability to produce insulin due to the progressive and selective destruction of beta cells in the pancreatic islets of Langerhans by the immune system [20]. A reduction in endogenous insulin production results in an increase in plasma glucose (hyperglycemia). Chronic hyperglycemia exposes patients with type 1 diabetes to an increased risk for death if left untreated. Pathogenesis of the disease has been attributed to a variety of environmental and genetic risk factors [21]. Yet, two of the most significant challenges facing the clinical management of this disease is the increase in incidence of type 1 diabetes mellitus across the globe [22] and the lack of a cure.

One of the persistent challenges with understanding the etiology of type 1 diabetes mellitus is the inability to observe directly the events in the human pancreas that lead to the onset of hyperglycemia. It is clear that a reduction in endogenous insulin production precipitates the onset of hyperglycemia. It is common wisdom that the onset of hyperglycemia occurs when 80–95% of an individual's beta cells are destroyed [23, 24]. However, this wisdom is based largely on a small number of biopsy studies from individuals with recent disease onset who died soon after diabetes onset (e.g., [25–27]). One might infer from this common wisdom that the ability to enhance beta cell function or preserve the remaining beta cells would have a limited therapeutic potential [28]. As a result, the research effort has focused on developing prognostic tools for identifying individual, who will develop type 1 diabetes, prior to onset. Given the clinical importance of this question, the objective of a recent study [29] was to develop a mathematical model to test the conceptual model for the pathophysiology of type 1 diabetes mellitus against the histopathological evidence.

**Figure 3:**
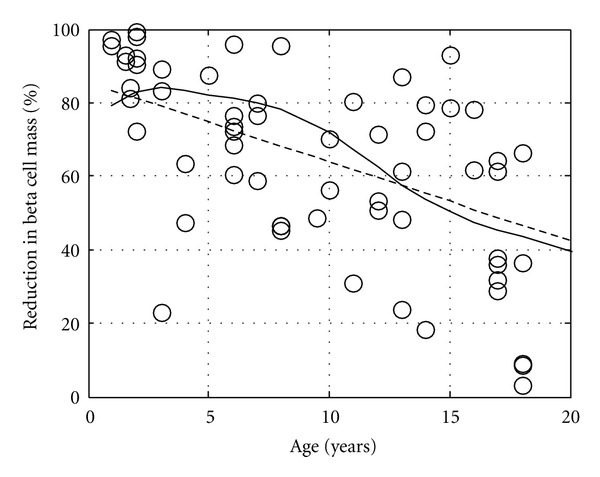
Comparison between the predicted and measured excess beta cell mass. Comparison of the excess beta cell mass predicted by the mathematical model (solid curve) compared against the trendline obtained by linear regression (dotted line) for the measured reduction in beta cell mass in 63 patients that died within three weeks of diagnosis of type 1 diabetes mellitus. Figure was originally published in [29].

A meta-analysis was used to extract and assess the significance of embedded trends within these landmark studies. The data reported in these landmark studies provide measurements of the remaining beta cells (i.e., beta cell mass) at the time of death. Patients included in these studies died between 0 and 69 months following diagnosis. While beta cell mass or endogenous insulin production is not measured directly following onset, C-peptide is used as a surrogate measure of endogenous insulin production [30–32]. The measurement of C-peptide in a cohort of patients with type 1 diabetes has been shown to vary nonlinearly with time following onset. In the years subsequent to onset of type 1 diabetes, the beta cell mass slowly declines until there is no endogenous insulin production. Therefore, inferring the beta cell mass at onset must control for this variability in the time of beta cell mass measurement. In this new analysis, the length of time following diagnosis was controlled by limiting the analysis to a subset of patients who died within three weeks following diagnosis. As shown in Figure 3, a linear regression of this subset of recent onset patients (dotted line) revealed that the percent reduction in beta cell mass at onset is not fixed but varies with age. This trend is significant (*P* < 0.01) and suggests that, in a 20-year old individual, as little as a 40% reduction in beta cell mass is sufficient to precipitate clinical symptoms of type 1 diabetes. As this trend is at odds with the existing model for the natural history of the disease [21], a mathematical model was created to explain this behavior [29].

The mathematical model was based on the observation that the growth of the human body is a dynamic nonlinear process where different parts of the body grow at different rates. Of particular relevance to type 1 diabetes mellitus, body weight changes [33] at a different rate than beta cell mass [27]. One possible explanation for this observed trend in extent of reduction in beta cell mass at onset could be attributed to the dynamic imbalance between the number of beta cells and the insulin requirements for a growing body.

**Figure 4:**
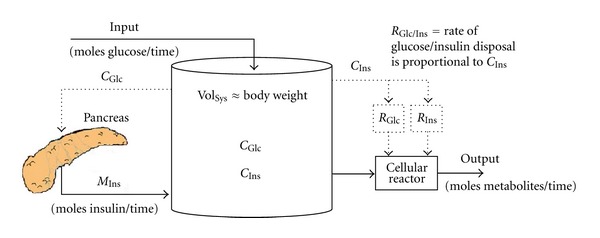
A schematic process diagram of the interplay between insulin production and glucose homeostasis. The regulation of substrate metabolism is modeled as an open system, where the concentrations of insulin (*C*_Ins_) and glucose (*C*_Glc_) are influenced by flows in (e.g., a glucose input and pancreatic production of insulin (*M*_Ins_)) and out (e.g., the disposal of glucose through cellular metabolism or the disposal of insulin through cellular proteolysis) of the system. Material flows are represented by solid lines while the flow of information is represented as a dotted line. For instance, the production of insulin by the pancreas—a material flow—is regulated by concentration of plasma glucose-a flow of information. The molar production of insulin by the pancreas is the product of the beta cell mass times the insulin production per beta cell. Similarly, the rates of disposal of insulin and glucose within the system (*R*_Ins_ and *R*_Glc_) are regulated by the concentration of plasma insulin. The concentration of insulin is a derived intrinsic quantity where the moles of insulin produced by the pancreas are distributed throughout the system volume, which is proportional to body weight.

**Figure 5:**
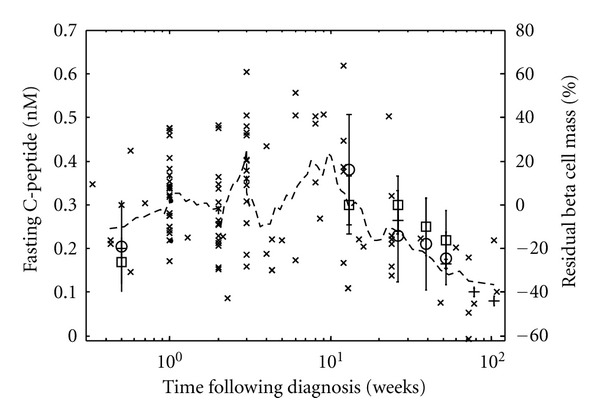
Dynamic change in residual beta cell mass corresponds to the dynamic change in plasma C-peptide following onset of type 1 diabetes. The residual beta cell mass (x: right axis) and plasma C-peptide (square [31], circle [32], and + [30]: left axis) are shown as a function of time following clinical diagnosis of type 1 diabetes. A 9-point moving average of the residual beta cell mass is shown for comparison (dotted line). The residual beta cell mass is the difference between the observed beta cell mass and predicted beta cell mass. The dynamic change in observed beta cell mass was obtained from pancreata obtained from patients with type 1 diabetes [25–27]. The predicted beta cell mass is an estimate of the minimum beta cell mass required to maintain glucose homeostasis. Figure was originally published in [34].

A mathematical model was used to predict the “excess” beta cell mass (EBCM) as a function of age by capturing the dynamic balance between changes in body weight and beta cell mass. The “excess” beta cell mass corresponds to the reduction in beta cell mass that is required before hyperglycemia occurs and is directly related to the measurements obtained in these landmark studies. This model, shown schematically in Figure 4, is derived from a mass balance on insulin and has a single adjustable parameter. Applying a mass balance to a system of interest is a common theme woven throughout the chemical engineering curriculum. In this instance, the rate of change in insulin is equal to the source of insulin, which is proportional to beta cell mass, minus the sinks for insulin, which are proportional to body weight [29]. The resulting model prediction for EBCM as a function of age is shown in Figure 3 (solid line). The trendline obtained by linear regression (dotted line) and the observed reduction in beta cell mass in pancreata obtained from the subset of recent onset patients (i.e., died within three weeks of diagnosis) are also shown for comparison. The EBCM relationship exhibits a similar dependence with age, as the youngest patients exhibited an 85% reduction in beta cell mass while only a 40% reduction was observed by the age of 20. In other words, the beta cell mass initially grows at a faster rate relative to the whole body. The beta cell mass peaks at 8 years of age and remains constant while the overall body weight peaks at 20 years of age. The net result of the different growth dynamics is that the “excess” beta cell mass declines with age. In addition, the mathematical model provides a prediction of the beta cell mass required to maintain glucose homeostasis. As a validation of the model, one finds that the difference between the observed and predicted beta cell mass (i.e., residual beta cell mass) parallels the observed changes in C-peptide following diagnosis (see Figure 5), as described in [34].

In summary, this model (i.e., prototype) suggests that clinical presentation of the disease is not attributed solely to the destruction of beta cell mass but is the result of a dynamic balance between the production of insulin (i.e., beta cell mass) and the size of the system (i.e., body weight). The agreement between the model-based predictions and the reported changes in C-peptide suggests two points. First, the methods that were used in these landmark studies exhibit a certain degree of accuracy in estimating beta cell mass, while the methods may not have had good precision. By using a mathematical model to interpret the trends in the data, we are able to correct for the imprecision of the assays used. Second, the similar dynamic trends suggest that the natural history of the disease is similar across the collection of clinical studies. While the biological details associated with the autoimmune attack on the pancreas and regulation of human metabolism are missing in this simplified model, the model exhibits a fitness for use in that it is sufficiently complex to answer the question posed. Using a mathematical model to represent our prior knowledge of the biology, the model provides a unique perspective to interpret these landmark studies which challenges the common wisdom in the field of type 1 diabetes. Improved understanding of the natural history of the disease—as it helps suggest causality—is a necessary prerequisite for improving the clinical management of the disease. Understanding causality is essential for developing new drugs that hold promise for a cure.

## 4. Example 2: The Role of Dendritic Cells in**** Adaptive Immunity

 The human immune system provides the body with natural defenses against the constant onslaught of overt and opportunistic pathogens. This defense against invading pathogens is an emergent behavior of a collection of heterogeneous cell subsets and typifies a complex system [35]. Individually, each of these subsets have unique roles in orchestrating an immune response. Together, these cell subsets integrate information across a range of spatial and temporal timescales. Despite the impressive advances in the field of immunology in the past decades, we know relatively little about the interplay between the individual components responsible for immunity [1, 36]. A mathematical model provides a quantitative framework where fragmented knowledge can be synthesized to predict integrated behavior of these components. In the remainder of this section, we will discuss a prototype that focuses on a cell subset that plays a central role in orchestrating an immune response—dendritic cells (DC)—in the lung.

**Figure 6:**
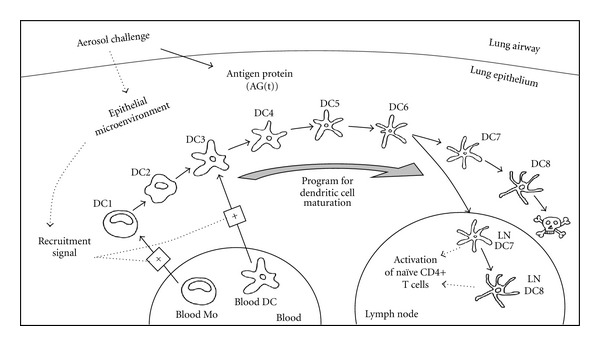
Schematic diagram of mathematical model for dendritic cell trafficking within the lung epithelial microenvironment. An aerosol challenge results in the increase of antigenic proteins and the recruitment of DC precursors from the blood into the lung epithelium. DCs dynamically traffic through the lung epithelium and become programmed by the prevailing epithelial microenvironment. Upon maturation, DCs migrate into the lymph nodes and present antigenic peptides obtained in the lung epithelium to naïve CD4+ T helper cells.

As the sentinels of the immune system, dendritic cells (DCs) play an important role in initiating and maintaining T-cell responses, such as T-helper cell polarization and crosspresentation of exogenous antigens to cytotoxic T cells [37, 38]. The precise role played by DC in *de novo* activation of T cells is the culmination of a series of steps distributed across both space and time. These sequential steps include the recruitment into a peripheral tissue, capture of antigen, trafficking to a draining lymph node, and presentation of antigen to T cells [37, 39]. A generalized schematic of this process is shown in Figure 6. Human biopsy data suggest that the majority of dendritic cells in the lung epithelium are derived from either blood monocytes (BMs) or blood dendritic (BD) cells [40]. Individually, BM and BD represent 97% and 3% of the DC precursor population in the blood. Although these DC precursor cells can be easily assayed in the blood, their relative contributions to the dendritic cell population within the lung epithelium and their functional roles in driving an immune response are unknown. Moreover, the role of BD has been largely ignored due to its relative rarity as a DC precursor.

To explore the implications of DC precursor recruitment into the lung, we created a mathematical model that captures the dynamics and origin of tissue dendritic cells [41, 42]. The dynamic model suggests that BDs are selectively enriched within the lung as they comprise 20% of the DC population in the lung [41]. While it is intriguing that BD may exhibit a higher affinity for the recruitment stimuli compared to BM, a more important question is whether this observation is functionally significant. The structure of the model was designed to capture an important aspects of dendritic cell biology—an age-structure.

As a dendritic cell traverses from blood to lung to lymph node, it turns on different “subroutines” encoded within its genes enabling it to perform different functions within each compartment. The dynamic execution of these subroutines is represented by dynamic changes in proteins expressed on the surface of a DC. The sequence of cellular changes are collectively referred to as DC maturation. Proteins expressed on the cell surface enable a cell to sense and respond to its environment. These dynamic changes in DC proteins indicate that the particular cellular response of a DC to the environmental context is highly dependent on the DC's particular maturational age. In addition, the ability of a DC to capture and process protein antigens derived from invading pathogens is also highly dependent on the maturational state of a DC. Given the dynamic nature of the DC population, the appropriate computational paradigm for representing DC populations is a model structured by maturational age [41, 42].

While physiologically-structured models have been proposed since the mid-1960s [43], they are seldom used to describe cell populations due to the difficulty in obtaining appropriate experimental data and the mathematical complexity of the resulting models. Given appropriate data, the additional complexity enables asking different questions. The dynamic response of cell populations in the blood to perturbations has been represented using physiologically-structured models (e.g., [44, 45]). In this case, age-associated differences in antigen processing ability of these two DC precursor populations can be compared by explicitly tracking the functionally unique subpopulations. Differences between BM- and BD-derived DC become especially apparent when antigen proteins also change with time. When antigen proteins have a half-life in the tissue of 60 minutes, BD-derived DC presents 250% more antigen peptide per cell relative to the DC derived from BM. *De novo* activation of T-helper cells requires that signals, including the density of antigen peptides, exceed activation thresholds [46, 47]. If the density of antigen peptides is averaged across all DC subsets, the dynamic change in density of peptides may be below the threshold required for activation of T-helper cells. By explicitly accounting for variability in DC phenotypes, the density of peptides presented by this minority DC subset may exceed the threshold for activation. While these studies highlight the importance of measuring DC heterogeneity, they also highlight how computer models can be used to integrate heterogeneous data into a quantitative picture of the dynamic role of dendritic cells in coordinating immunity.

## 5. Reflecting Back: Goldilocks and the Two Maxims

 The use of models to aid in understanding system behavior is a central theme in science that transcends disciplinary boundaries [48]. In the previous sections, two examples served to illustrate some of the nuances associated with mathematical modeling from an engineering perspective, namely, the concepts of prototyping and fitness for use.

In the case of the type 1 diabetes model, the two competing theories are that the degree of beta cell reduction at onset is a fixed value or that the observed reduction is a result of a dynamic balance between beta cell mass and body weight. From a mathematical perspective, the models exhibit similar complexity as both models use a single adjustable parameter to predict the observed behavior. The Akaike Information Criterion [49–51], based upon information theory, is used to distinguish between these competing models using the available data. Intensive computing techniques, like nonparametric bootstrap resampling [52, 53] and empirical Bayesian methods [17], complement information-theoretic metrics by assessing the uncertainty of those metrics, given the inherent uncertainty in measuring biological systems. Moreover, this simplified model was also able to compare changes in beta cell mass to changes in C-peptide following diagnosis [34]. Finally, one could construct a model that includes more detail regarding the different timescales for insulin production [54–56], insulin signaling [57], and beta cell autoimmunity [58]. However, the simplified model exhibits a fitness for use as inclusion of such detail is unnecessary to test the prevailing theory.

In the case of the dendritic cell trafficking study, a new model is proposed to represent cellular heterogeneity and to provide an estimate of its potential importance. In contrast, existing models that assume that all dendritic cells are homogeneous (e.g., [59]) are unable to capture with the observed dynamic patterns of cell surface marker expression during dendritic cell maturation [60–62]. Additional structure is required to represent this cellular heterogeneity within the model. Using computational techniques, such as parameter identification [63, 64], the increased cost, in terms of parameters, associated with a more complex model that captures a larger set of data is justified. Yet, the age-structured modeling framework is not well suited to explore questions related to the spatial organization of the lymph node or the discrete nature of cell-to-cell interactions. The form of the age-structured model, which is a set of coupled ordinary differential equations, assumes that the age compartments are well mixed, that is the cells are homogeneous within an age compartment. Agent-based models of the lymph node are better suited to such questions [65–68]. Historically, agent-based models focus on cell population-level behavior and neglect the molecular details associated with cellular decision making, such as an evolution in cell phenotype due to local changes in developmental cues. Although, models that aim to combine cellular-level with population-level behavior are emerging [69]. This highlights the iterative nature of the engineering design process. As additional data become available, the mathematical prototype can be revised to reflect this new information. Moreover, the form of the model may change depending on the fitness for use of the particular mathematical framework (e.g., ordinary differential equation-based or agent-based model) to address the questions of interest.

Reminiscent of the notable children's story “Goldilocks and the Three Bears,” a common criticism of a particular mathematical model is that it is either too complicated or too simplistic. In many cases, these statements are subjective as they are based upon the collective experience of the critic [19]. One of the benefits of representing theory in a computable form is that computational tools can be used to assess objectively the complexity of the model. Implied in the criticism is the question of model parsimony. Conventionally, there are two maxims that bracket the range of plausible explanations for observed phenomena: Ockham's Razor and Einstein's Safety Shield. The concept of Ockham's Razor is that if there are a series of theories and the available data cannot distinguish between the different theories, then the simplest theory should receive priority. The concept of Einstein's Safety Shield is that one should construct the simplest theory to explain observed phenomena but no simpler. The emergence of information-theoretic approaches provides a quantitative basis for these maxims (see [70] for an introduction to the topic). While these are important topics to consider when modeling immunology [71], information-theoretic concepts have been infrequently applied to modeling efforts in the field [72]. Recent developments in rule-based modeling [73–75], time scale analysis [76], and in silico model-based inference [17] all help reduce the barrier for integrating theory—in the form of mathematical models and engineering concepts—with experimental immunology. Within the domain of cellular decision making, the combination of these three modeling developments allow one to specify a mathematical model with limited a priori bias in the model structure and use the available data to determine objectively the appropriate level of complexity, as illustrated in this sequence of papers [76–78].

In summary, engineering is historically a field in which basic research is translated into commercially viable products and processes. The commercial synthesis of basic science data is achieved using computational frameworks. Translating data into knowledge is a major challenge facing contemporary health science research. Two examples discussed in the previous paragraphs aim to illustrate how the computational toolkit of an engineer can be integrated into experimental immunology via mathematical prototyping. These examples also serve to illustrate that embracing a quantitative perspective provides an opportunity to integrate focused experimentation into a larger mosaic that describes human immunity. Through mathematical prototyping we are able to represent explicitly our prior knowledge of the dynamics of immunity and test this prior knowledge against experimental data. Moreover, the process of creating a mathematical model provides a roadmap for future experimental effort by identifying important knowledge gaps in the collective scientific understanding. Ultimately, improved understanding of the complexity of biological systems is essential for promoting human health and restoring health through the rational design of new therapeutics.
